# Improving relationship-centered care through evaluation meetings with the resident-family-caregiver triad in nursing homes: a qualitative study

**DOI:** 10.1186/s12913-025-12425-1

**Published:** 2025-02-22

**Authors:** Johanna E. R. Rutten, Ramona Backhaus, Hilde Verbeek, Erica de Vries, Jan P. H. Hamers, Katya Y. J. Sion

**Affiliations:** 1 Department of Health Services Research, Care and Public Health Research Institute, Maastricht University, Maastricht, The Netherlands; 2 Living-Lab in Aging and Long-Term Care, Maastricht University, Maastricht, The Netherlands

**Keywords:** Relationship-centered care, Quality improvement, Care triad

## Abstract

**Background:**

Providing and improving relationship-centered care has gained increased importance in long-term care. However, quality improvement strategies are predominantly based on quantitative quality measures for care professionals. Therefore, the aim of this study was to explore how narrative data collected with Connecting Conversations is used in evaluation meetings to improve RCC.

**Methods:**

A qualitative approach using structured observations was used. The participants were care professionals, residents and family members from two nursing home wards. The participating wards organized evaluation meetings to discuss the experienced quality of care based on narrative data collected with Connecting Conversations. To analyze the process of improving RCC, the organization of these meetings and the content were observed by independent researchers, and detailed notes were taken. The data were analyzed thematically by using conventional content analysis.

**Results:**

In total, three evaluation meetings were organized. Primarily, care professionals were invited to discuss the results of the interviews. One ward organized a meeting for care professionals, residents and family members, and the other decided not to invite them. The discussion of themes related to experienced quality of care within the evaluation meetings was less profound than during the interviews. In total, 12 overarching themes concerning experienced quality of care were discussed in the Connecting Conversations' interviews. Nine themes were also mentioned in one or more evaluation meetings (i.e., activities for residents, personalized attention and preferences of residents, feeling at home and communication within the care triad).

**Conclusion:**

When using narrative data on quality of care to improve relationship-centered care, the full potential of narrative data is underutilized as discussions focussed on incidental problem solving rather than deeper reflections on the meaning of events in providing relationship-centered care. Establishing trust within the care triad of care professionals, family members, and residents is essential to improve relationship-centered care collaboratively.

## Background

Nursing homes provide long-term care for frail and older adults with complex health conditions. People who receive long-term care in a nursing home are likely to stay for a longer period of time or even for the rest of their life [36]. To experience a good quality of life after moving to a nursing home, feeling at home is fundamental. This feeling develops from the interactions an individual has with the environment in which he or she lives [30]. The social networks and relationships of nursing home residents can also contribute to feeling at home [13].

In recent years, the concept of providing relationship-centered care (RCC) in nursing homes has gained increased importance [41]. RCC acknowledges the importance of relationships within the healthcare process and views the individual as part of his or her personal relationships [8, 41]. RCC is based on four principles: first, that personhood matters; second, that affect and emotion are important; third, that relationships do not occur in isolation; and, fourth, that maintaining genuine relationships is necessary for health and recovery and is morally valuable [8]. Furthermore, RCC recognizes the perspectives of all involved stakeholders in the care process [33]. Henceforth, in the nursing home setting, it addresses the needs of residents, their family members and care professionals, and can thereby improve the experienced quality of care of the care triad [31, 46]. RCC recognizes individuals as active participants in their interactions and takes into account the relationships, diverse needs, desires, and perspectives that emerge throughout the care process [8, 31].

In long-term care, the provision and improvement of RCC has become an important quality indicator of high quality of care [41]. Until recently, most quality improvement strategies in nursing homes have been based on quantitative quality data that represent clinical outcomes such as the number of falls, pressure ulcers or pain [43]. One commonly used measurement for such quality improvement strategies is the patient-reported outcome measure (PROM). Studies on the use of PROMs as quality improvement instruments show that care professionals value the use of these data for implementing quality improvement processes [10]. Strategies that focus on specific issues at the individual resident level, such as disease progression, seem to be effective [9].

In order to enhance the effectiveness of using data for quality improvement, it is recommended that the residents’ perspectives are included when acquiring feedback. This approach could facilitate the possibility of linking data to an individual resident level. It is also expected that data are more valuable for care professionals when they can be linked to the individual resident level [10]. To prioritize different perspectives in the use of quality data, the narrative method ‘Connecting Conversations’ could be used. Connecting Conversations is based on the ‘Indexqual framework’, in which experienced quality of care (QoC) is conceptualized as a process influenced by events occurring before, during and after a care experience [39]. It places RCC at the core of all actions taken within the care process. Connecting Conversations assesses experienced QoC by having separate appreciative conversations with residents, family and caregivers, the so-called care triad. Furthermore, it is assumed that data collected with Connecting Conversations can be used to introduce quality improvement strategies within the care triad. The narrative data provide insights into all three perspectives of the care triad and a rich understanding of what is going well and what could be improved [38]. Narrative data have been shown to be valuable for providing insights into a person’s experiences and displaying an individual point of view on a specific topic, such as experienced quality of care [49]. These insights can be used to stimulate a quality improvement process. Narratives offer the opportunity to include different needs, experiences and perspectives on QoC in an improvement process [18, 24]. Earlier studies have shown that care professionals are motivated to use narratives to improve the QoC, as they portray the experiences of residents, their families and care professionals in everyday practice [45].

However, additional knowledge is needed on how narrative data could be used for quality improvement. Research has shown that a structured approach for translating narrative data into quality improvement strategies is necessary for effective implementation [15, 16]. Additionally, research suggests that leadership behavior is an important factor for the successful implementation of quality improvement strategies [20, 50]. Furthermore, quality improvement interventions mostly consider specific target groups instead of fostering collaboration between different stakeholders. For instance, interventions intended to improve family inclusion in nursing homes often consider only care professionals as a target group without including family members in the process [3]. However, to adopt an effective quality improvement process, it seems necessary to involve the care triad in the whole learning process [25]. Therefore, the aim of this study was to explore how narrative data collected with Connecting Conversations are used in evaluation meetings to improve RCC.

## Method

### Design

For the purpose of this study, a qualitative approach was chosen, consisting of structured observations during evaluation meetings conducted in March and September 2022.

### Setting and participants

The sample consisted of two nursing home wards located in the south of the Netherlands. In the Netherlands, nursing homes provide short-term rehabilitation care and long-term care for people with dementia or serve disabilities within psychogeriatric wards or somatic wards. A somatic ward in a nursing home refers to a specialized area or unit designed for residents who require care for physical (somatic) health conditions, rather than cognitive or mental health conditions. Direct care is mostly provided by certified nurse assistants who have followed a 3-year educational program. Furthermore, direct care teams in Dutch nursing homes also consist of lower educated professionals, such as nurse assistants who have followed a 2-year training and nurse aides who have followed a training between 0.5 and 1 year [3, 4]. These direct care teams collaborate with vocationally trained or baccalaureate-educated registered nurses, medical specialists and associated health professionals (e.g., physiotherapists, psychologists, occupational therapists).

For this study, one psychogeriatric ward and one somatic ward were included. Nursing homes were participating in the Living Lab in Aging & Long-Term Care Limburg the Netherlands, a structural collaboration of four educational institutions and nine long-term care organisations [48]. The teams consisted of care professionals working at the ward, residents living at the ward and family members. The inclusion criterion was that the wards performed a quality measurement according to Connecting Conversations.

### Data collection

First, the narrative method Connecting Conversation interviews were conducted. Connecting Conversations is a narrative method in which separate semi-structured in-depth interviews are conducted with residents, their family members and care professionals. The interview guide consisted of six main questions about the experienced quality of care. The interviewers were employed or connected to a healthcare organization and received three days of training to perform the interviews. All interviews were audio recorded, and participation was voluntary. Detailed information on this method has been published elsewhere [38]. Second, after completing the Connecting Conversations interviews, participating teams organized meetings on their own initiative to evaluate the results of the Connecting Conversations interviews. All data from Connecting Conversations was initially analyzed by a researcher. Participating teams were responsible for the organization and participant selection of the meetings. Furthermore, teams were free in designing the presentation of the results and providing feedback. The results of the Connecting Conversations interviews were the main input for each evaluation meeting.

During all the evaluation meetings, detailed notes were taken by an independent researcher, whose role was explained beforehand to the participants. The notes were structured according to an observation guide, which is presented in Table 1. The observation guide was based on factors for improving RCC mentioned in the literature (i.e. leadership behavior, organizational circumstances) [20, 42]. Third, detailed notes were taken during informal conversations with all stakeholders involved in the process of organizing the meetings.

**Table 1:** Observation guide

Context	- Date of the meeting - Duration of the meeting - Type of participating ward - (invited) Participants
Type of meeting	- Location of the meeting - Aim of the meeting - Setting of the meeting (e.g., regular team meeting)
Dynamics during the meeting	- Who is the facilitator of the meeting? - Do all participants actively participate during the meeting? - Who does speak up?
Role of the manager	- Is the manager present during the meeting? - What is the formal role of the manager during the meeting? - Which activities does the manager perform during the meeting?
Content of the meeting	- Which themes are discussed during the meeting? - Which (new) agreements/ improvement points are made during the meeting? - How is the allocation of responsibilities and the timeline made? - Is there an evaluation of the meeting?
Additional comments	

### Data analysis

First, conventional content analysis of the Connecting Conversations interviews was performed by a researcher to compare relevant themes concerning experienced QoC with themes discussed during the evaluation meetings. Conventional content analysis is suited for describing a phenomenon on which theory and earlier research are limited, because of its inductive approach [23]. This way of analyzing allows new insights to emerge from data by avoiding preprepared categories [27].

Second, detailed notes from the evaluation meetings and informal conversations were analyzed. The context (i.e., setting and participants) and process of organizing the evaluation meetings were described. To capture themes, conventional content analysis was applied. All the actions that were intended to improve RCC were thematically coded and summarized in themes.

Third, the themes emerging from the evaluation meetings were compared to the themes emerging from the Connecting Conversations interviews concerning the experienced QoC on the ward.

The codes and central themes were discussed within the research team, which consisted of researchers with different backgrounds (i.e., psychology, nursing and health sciences). Throughout the whole process of data analysis, differences were discussed within the team, resolved and adjusted. The data were analyzed with MAXQDA version 22.2.1 software.

### Rigor

Different strategies were applied to enhance the rigor of this study. Triangulation of the data was applied by using structured observations and (informal) interviews at different times and from different people to capture different aspects of the findings (e.g. why family members were not invited) and to enhance completeness [17]. The coding frame was refined by cross-checking the coding process and the clustering of the data within the research team [6]. A systematic approach was used for the observations to enhance reliability. Furthermore, continuously reflecting on the role of the researchers in the process of data analysis and interpretation was applied to enhance reliability through reflexivity [26].

## Results

First, the process and structure of the evaluation meetings are described. Hereafter, the content of the evaluation meetings is described and compared to the content discussed in the Connecting Conversations interviews. In total, 10 care professionals, 10 residents and 10 family members from two wards participated in the Connecting Conversations interviews (see Table 2).

**Table 2:** Characteristics of the wards

	**Ward A**	**Ward B**
Type of ward	Somatic	Psychogeriatric
Number of residents living on the ward	27	8
Number of care professionals^a^ working on the ward	34	10
Number of Connecting Conversations’ interviews	6 triads (18 interviews) Residents: *N* = 6 Family members: *N* = 6 Care professionals: *N* = 6	4 triads (12 interviews) Residents: *N* = 4 Family members: *N* = 4 Care professionals: *N* = 4

^a^All staff involved in daily care of the residents

Three evaluation meetings, planned and organized by the healthcare organizations, took place. A fourth meeting was planned but did not take place due to the withdrawal of the participants (family members and residents). Participants were 23 care professionals, three family members, two residents and two managers. The results showed that the wards organized the evaluation meetings differently regarding the setup of the meetings, but both wards primarily invited care professionals to attend the meetings. Ward A organized an additional meeting with family members and residents, whereas ward B decided not to invite family and residents at all. The duration of the meetings differed among the meetings and wards. The meeting on ward A with care professionals lasted 30 min and occurred during a regular team meeting, and an additional meeting was scheduled with care professionals, residents and family members, which lasted 120 min. Ward B was assigned 90 min in an extra scheduled meeting. The main results show that more themes are discussed during extra scheduled meetings than during an evaluation meeting incorporated in a regular team meeting. Table 3 presents an overview of the characteristics and the participants of the evaluation meetings. First, the process and structure of the evaluation meetings and collaboration with the care triad will be presented, followed by the content of these meetings.

**Table 3:** Characteristics evaluation meetings

	**Ward A**	**Ward B**
Meeting 1		
Facilitator	Researcher (JR) and manager (*n* = 1)	Internal quality assurance employee (*n* = 1)
Participants	Care professionals (*n* = 14);	Care professionals (*n* = 5); manager (*n* = 1), research assistant (*n* = 1)
Note taker	Research assistant (*n* = 1)	Research assistant (*n* = 1)
Setting	Regular team meeting	Extra planned team meeting
Duration	30 min	90 min
Meeting 2		
Facilitator	Informal caregiver supporter	
Participants	Residents (*n* = 2), family members (*n* = 3), care professionals (*n* = 4),	
Note taker	Researcher (JR) (*n* = 1)	
Setting	Informal meeting	
Duration	120 min	

### Process and structure of the first evaluation meetings

On ward A, the manager appointed a regular scheduled team meeting for all care professionals of the ward to discuss the findings emerging from Connecting Conversations. The manager designated 30 min of the meeting for the presentation of the results and discussion. To maximize the impact of the findings for the care professionals, the manager requested that an independent researcher (JR) present the results. The researcher prepared a presentation consisting of a summary of the main results and anonymized quotes from one of the care triads (interviews with residents, family members and care professionals) to display how the interviews were performed and analyzed. It was a conscious decision of the manager not to invite family members and residents to this meeting because the detailed results were, according to him, not suitable to share with family members and residents.

The manager led the conversation during the meeting and encouraged the care professionals to discuss the results by asking them questions (i.e., “what do you think about this?”). During the meeting, the manager was the most active participant because he or she reflected on the results and tried to involve the rest of the participants by discussing the results. The care professionals identified only one improvement point during the discussion. This improvement point was that they should work more collaboratively with family members and clearly communicate their expectations. They expressed that the presented results were too globally formulated, which impeded the formulation of improvement points. According to them, it is necessary to know which narrative belongs to which resident. No further improvement points were formulated during the meeting.

“You don't know which resident it is [the interview] about. If you don't know who it is, you can't work on it. That's a pity” (Care professional, ward A).

On ward B, an evaluation meeting was organized by an internal quality assurance employee, who served as a facilitator and led the discussion. The meeting was scheduled as an extra meeting to discuss the results of the Connecting Conversations interviews, and all care professionals from the ward and the manager were invited to attend. The facilitator aimed to achieve active engagement of the participants by including them in the analysis of the findings.

Therefore, the manager of the ward was tasked with appointing two care professionals to read and summarize the results of the interviews, identifying at least three tips (what can be improved) and tops (what is going well), which were included in the presentation. All participants participated actively during the meeting by expressing their opinions on the presented results. Participants mentioned that an extra scheduled meeting increased the workload and that they would prefer the evaluation meeting during a regular scheduled team meeting. They appreciated how they received feedback with the tips and tops and emphasized that they would also like to read the full interviews. There was no follow-up meeting planned with the same participants after the meeting because, according to the facilitator, themes concerning RCC mentioned in the Connecting Conversations interviews will be frequently discussed in the future during quarterly meetings to follow-up improvement processes.

“[It is] Great that a limited number of people prepare tips and tops. It is preferable that an independent person does it (also).” (Care professional, ward B).

### Process and structure of the second evaluation meetings

On ward A, a second meeting with residents, family members and care professionals was organized by the informal caregiver supporter. The informal caregiver supporter, hereafter called the facilitator, was employed at the nursing home, and the ward manager assigned her to facilitate and improve collaboration with family members. The facilitator got to know all the invited family members in advance by having individual conversations with them. A selection of care professionals, residents and family members was invited. During the invitation process, the facilitator experienced that family members were not motivated to attend an evaluation meeting. In total, five family members accepted the invitation, but two cancelled the last minute. Two residents were willing to participate during the meeting.

The meeting was organized to discuss and improve collaboration within the care triad. It was a conscious decision to not invite the manager for this meeting to create an informal and safe atmosphere. The facilitator received the results of Connecting Conversations to prepare for the meeting and used the information to guide the conversation. All care professionals and family members participated actively during the meeting by sharing their views and experiences with the rest of the group. Residents remained passive during the meeting. The facilitator stimulated the conversation and summarized the discussed themes. All participants agreed on one improvement point aimed at better collaboration within the care triad. This improvement point consisted of an informal Easter lunch for residents, family members and care professionals organized by one care professional and one family member.

In addition, the facilitator evaluated the meeting with the participants at the end. Participants appreciated the open conversations with the other attendees and the possibility of getting to know each other in an informal setting. Participating care professionals indicated that the evaluation meeting made them aware of the fact that the collaboration with the family could be improved.

“[The evaluation meeting was] valuable; we can learn a lot from each other, this creates some awareness” (Care professional, ward A).

A follow-up meeting was planned by the facilitator with all participants during the evaluation meeting, but all family members cancelled their attendance at this meeting. The family member who was appointed to organize the informal Easter lunch withdrew shortly after the evaluation meeting, and the lunch did not take place. In an informal meeting with the manager and the facilitator afterwards, the manager highlighted that, according to him, family members might be too busy taking care of the residents to spend time on general quality improvement initiatives. The facilitator mentioned that family members might be satisfied with the delivered care and therefore do not see the urgency to improve it.

On ward B, the facilitator explained that it was a conscious decision to not invite family or residents to the meeting to avoid possible conflicts that would emerge when putting these stakeholders together. Therefore, no second meeting including residents and family members was planned.

“We were “afraid” that the discussion would go in the wrong direction, if we invite family and residents to the meeting, because then you have different positions at the same table” (internal quality assurance employee, ward B).

### Comparison of the content of the evaluation meetings with the content of the Connecting Conversations interviews

The content of the interviews and the evaluation meetings were summarized in overarching themes that arose from the data and are considered relevant regarding RCC. In total, 12 themes were discussed in the Connecting Conversations interviews, nine of which were also mentioned in one or more evaluation meetings (Table 4).

**Table 4:** Comparison of themes mentioned as relevant to RCC in the interviews and the evaluation meetings

Themes related to relationship-centered care	Ward A			Ward B	
	**Mentioned in interviews**	**Actively discussed during meeting 1**^a^	**Actively discussed during meeting 2**^b^	**Mentioned in interviews**	**Actively discussed during meeting 1**^a^
Activities for residents	** + **	** + **	** + **	** + **	** + **
Personalized attention and preferences of residents	** + **	** + **			** + **
Feeling at home	** + **		** + **	** + **	** + **
Autonomy of the residents	** + **		** + **	** + **	** + **
Communication within the care triad	** + **	** + **	** + **	** + **	
Relationships within the care triad	** + **			** + **	** + **
(Family) Visit	** + **		** + **	** + **	** + **
Organization of work in the nursing home	** + **			** + **	** + **
Personal Care of residents	** + **			** + **	
Social contact of residents	** + **			** + **	
Life in the nursing home	** + **			** + **	
Improvement processes in the nursing home	** + **			** + **	

^a^Participants: care professionals, manager, researcher

^b^Participants: care professionals, Residents, family members, informal caregiver supporter, researcher

The content of all themes is summarized in Table 5. Themes that were discussed within the evaluation meetings were less profound in comparison to the same themes discussed during the interviews. The evaluation meetings remained rather superficial and focused more on specific events and how to solve these instead of discussing their deeper meaning for providing RCC. This is especially the case for the following themes: Activities for residents, Personalized attention and preferences of residents, Feeling at home, Autonomy of the residents and Communication within the care triad.

**Table 5:** Content of themes related to RCC within the evaluation meetings and the interviews

Themes related to RCC	Content of the interviews	Content of the evaluation meetings
Activities for residents	• Small activities which residents do in everyday life (i.e., going for a walk, helping in the kitchen) • Importance of activities to prevent inactivity of residents • Bigger events (i.e., parties and excursions) create positive memories for the residents	• Need for more general activities for residents (i.e., playing games) • Desire for more family involvement during activities with the residents
Personalized attention and preferences of residents	• Importance for care professionals and residents to take into account personal preferences of the residents • Importance of physical contact between residents and care professionals during for instance daily care activities • The way how care professionals try to take into account personal preferences in the care process • High workload and a lack of time as barriers	• Care professionals desire to give more personalized attention to the residents • Permanent staff is a preliminary condition for personalized attention
Feeling at home	• The possibility to feel at home and every behavior of the residents is tolerated • Family members describe the importance to maintain rituals with residents • Interior of the wards	• Interior of the wards • Organization of the wards
Autonomy of the residents	• Importance of the mobility of the residents • Capability of residents to carry out specific tasks on their own • Getting the opportunity to carry out daily tasks • Making own decisions • Being and feeling dependent on other people	• Importance of the opportunity to carry out daily tasks for the residents • Having the control over the small things in life
Communication within the care triad	• Importance of communication within the care triad for RCC • Communication about agreements between the different stakeholders • Information provision • Communication channels being use • The way different stakeholders approach each other • Verbal behavior between different stakeholders	• Information provision • Need for more informal information exchange between family members and care professionals
Relationships within the care triad	• Quality of the relationships between resident and family, resident and care professional and family and care professional • Importance of the relationships	• Expectations that family members have when their loved ones enter the nursing home regarding activities • Care professionals experience, that the expectations of family members are too high and not feasible
(Family) Visit	• Family involvement in the nursing home • Ways how family members spent their time with the residents • Effects of the family visits on the resident and the family • Restrictions due to the COVID-19 pandemic	• Possible family participation during activities
Organization of work in the nursing home	• Experienced workload of the care professionals • Work climate • Staffing and the organization of daily tasks	• General issues during care provision • Lack of staffing
Personal Care of residents	• Physical and medical care of the residents • Available time for physical and medical care • Safety of the residents • The way care professionals provide physical and medical care for the residents	• *Not discussed during the meetings*
Social contact of residents	• Effects that social contact between different residents and residents and care professionals have on the residents and loneliness	• *Not discussed during the meetings*
Life in the nursing home	• Statements which are given on how satisfied residents and family members are about the life in the nursing home in general	• *Not discussed during the meetings*
Improvement processes in the nursing home	• Activities which are taken to improve the quality of care • New ways of workplace learning and new ways of organizing daily tasks	• *Not discussed during the meetings*
		• RCC: relationship-centered care

#### Activities for residents

In all evaluation meetings, care professionals discussed activities (other than activities of daily living) for residents. These were mainly addressed as general activities and were mainly instrumental; i.e., the performance of the activity was the most important topic of discussion, not the aim or why activities matter to residents. During the interviews, the importance of activities and their effects were highlighted, such as making residents’ lives more convivial and preventing inactivity.

“Yes, that she [the resident] is kept a little more active. She is known as being very passive. […] you just see her not taking any initiative. If you put her in the chair at 8 or 9 o'clock in the morning, she stays there and does not come out.” (Connecting Conversations interview: family member, ward B).

#### Personalized attention and preferences of residents

In two evaluation meetings, care professionals superficially discussed their ambition to provide more personalized attention to the residents and the importance of considering residents’ personal preferences. They focused on the need for permanent staff (who are employed on a long-term basis) as a condition to do so. The importance and effects of physical contact for both care professionals and residents during daily care activities were not mentioned in the evaluation meetings. During the interviews, different ways to provide personalized attention were mentioned. A high workload and lack of time during daily activities were considered barriers.

“I think we also look specifically at her [the resident] wishes and try to make that happen, and when this is not possible, we are just honest about that as well.” (Connecting Conversations interview: care professional, ward B).

#### Feeling at home

In order to evaluate how residents develop or enhance a feeling at home on the ward, the interior and the organization of the ward were discussed during two evaluation meetings.

“My mother [the resident] always had a garden with a lot of flowers, that made her happy. Now she only has a few flowers in her room.” (evaluation meeting: family member, ward A).

It was not mentioned that feeling at home is also influenced by, for instance, the possibility for residents to behave as they would at home and that all behaviors should be tolerated and not restricted by rules. Furthermore, during the interviews, family members described the importance of maintaining rituals they had with their loved ones before they entered the nursing home to establish a feeling at home for them and the residents. These points were not addressed during the evaluation meetings.

“Yes, it makes one feel at home. Because there is also cooking here. If they [the residents] want, they can help peel potatoes.” (Connecting Conversations interview: family member, ward B).

#### Autonomy of the residents

During two evaluation meetings, the autonomy of the residents was discussed regarding daily tasks that residents could carry out or control small things in life. Other factors influencing the sense of autonomy of the residents were not discussed. In the interviews, the effects of losing control in everyday life activities, becoming dependent on other people, making one’s own decisions and being able to move around independently were mentioned.

“So yeah, well, she [the resident] turned out to have a[ medical condition]. So those are all things where she has had to give up more and more of her control. Yeah, she has a hard time with that sometimes.” (Connecting Conversations interview: family member, ward A).

#### Communication within the care triad

Communication was discussed during two evaluation meetings, which focused on providing information and the need for more informal information exchange between family members and care professionals. During the meetings, care professionals focused on methods of communication (how) rather than on the effects and importance of communication (why). The interviews also addressed how different stakeholders approach each other, how verbal behavior between different stakeholders and the effects that communication can have for the relationships within the care triad.

“It's mainly, of course, the care people are here for personal care, physical care, nutrition. However, to feel happy, you need more than that. And that little bit more is just not there. And that's very dependent then on the person, how empathetic they are to people, how kind they are to people. That's really different one to another. And I think it's unfortunate that personal approach towards the residents depends on the character of the people. I think there should be more attention to that from the policy.” (Connecting Conversations interview: family member, ward A).

## Discussion

This study aimed to explore how narrative data collected with Connecting Conversations are used in evaluation meetings to improve RCC. Participating teams used these narrative data to reflect on during the evaluation meetings. Our findings showed that evaluation meetings were primarily organized for care professionals. It was considered challenging to include family members and residents in reflecting on the results during the evaluation meetings. Regarding the content of these meetings, the results of our study highlighted that the narrative data were only superficially discussed, focusing on incidental problem-solving. The full range of details provided by the data was not used to reflect on RCC or to explore why certain themes were important to achieve RCC.

Whereas previous research has shown that care professionals are motivated to use narrative data, the findings of our study revealed that the use of narrative data for reflection by care teams requires additional guidance and structure [45]. A structured approach is recommended to use narrative data for quality improvement processes. To use the full range of details provided by narrative data, the preparation and setting of an evaluation meeting seem important. Meetings that are extra scheduled to discuss narrative data last longer and therefore offer more space to reflect on the data instead of discussing the results as one topic during a regular team meeting. In addition, reflection on QoC is part of care professionals’ daily work and should occur frequently, both informally and formally, to enhance a safe climate and reach depth together [5].

A possible explanation for the less profound discussion during the evaluation meeting could be that teams were not used to reflect on the results regarding RCC on their ward. Our data showed that care professionals mainly discuss themes concerning RCC superficially. The concept of experienced quality of care suggests that it consists of various factors, such as an individual's emotional state, expectations, and experiences [39]. Care professionals recognized the themes (what), for instance, the need for communication, but failed to discuss the importance or effect of communication on RCC. Within our study, care professionals tended to apply problem solving, as shown by the need to know which resident the data are about to “take action”. Argyris and Schön [1] refer to this as single-loop learning, where actions intend to correct mismatches. A deeper form of learning, so-called double-loop learning, considers variables that influence the results first before applying actions. The third and most profound way of learning mentioned in the literature is triple-loop learning, where results lead to an adjustment of the organizational context [44]. Earlier studies indicate that triple-loop learning requires a structured approach such as “set learning goals”, “monitoring” and “define learning action plans” [28].

Ideally, narrative data would help teams adjust their organizational context to improve RCC, but our results showed that teams are still mostly occupied with single-loop learning and might require a more structured approach.

Based on our data, it is not possible to conclude whether team members had the skills to reflect on RCC and formulate quality improvement strategies. Insufficient collaboration and weak relationships between team members could be possible explanations. Relational work, defined as the skills that ensure good relationships, is crucial for team reflection [5]. In addition, it is important to pay attention to the formation of a team to successfully implement quality improvement and learning strategies. Collaborative teams with longstanding relationships between team members are highlighted as facilitators of the implementation of QoC improvement strategies in nursing homes [14].

To learn and improve together as a team and to have a profound discussion, several prerequisites have been identified in the literature. A safe team climate and good team spirit can facilitate good communication and the opportunity to reflect together as a team [32]. It can be influenced negatively by changing team composition or when social support from a leader is missing [2 12]. In our study, social support for the care teams was provided by their ward managers, who were responsible for organizing data collection through the Connecting Conversations method and the evaluation meetings. Additionally, they played an active role in collaborating with the researchers. The literature suggests that leaders in nursing homes should participate and innovate to support quality improvements [11]. Furthermore, social support from a leader fosters a safe team climate, especially a transformational leadership style, which motivates care professionals to achieve organizational goals and has been shown to have strong links with improved quality of care [34, 37]. Therefore, it seems necessary that the leader supports the organization and implementation of evaluation meetings, but the role of a manager during such meetings should be further investigated.

Our results also showed that professional collaboration with the care triad does not occur automatically. When evaluation meetings are organized by care organizations, they primarily invited care professionals to the meetings instead of including residents and family members. However, when family members and residents were invited for participation, the results of our study showed that they are less willing to participate and withdraw easily. A possible explanation for this impeded collaboration could be a lack of trusting relationships and role ambiguity for care professionals and family members. To foster good relationships and collaboration between care professionals and family members, trusting relationships and clear communication about expectations and experiences with the provided care are crucial [22, 39]. The challenges of family involvement and collaboration between family members and care professionals are well studied topics. Care professionals often experience family members as “difficult”, too demanding and equal collaboration is burdensome [3 [7]. Family members struggle with power inequalities, communication issues and dependencies in collaboration with care professionals [19, 21]. The impeded collaboration between care professionals and family members during the evaluation meetings within our study showed that there might be a need to improve the relationship within the care field before anticipating a collaborative improvement process.

Our findings showed that limited residents were willing to attend the evaluation meetings and that the two residents who did attend these meetings remained passive. A possible explanation for this behavior could be that residents often feel dependent on their care professionals and are scared to jeopardize their relationship by expressing their opinions in front of them [46]. In addition, residents might be less capable of participating in these group discussions due to cognitive impairment or insufficient experience with quality improvement processes. However, research has shown that when nursing home residents have cognitive impairment and struggle to focus on a specific topic, sharing their values and preferences can still contribute to closer collaboration [35]. The severity of mild to moderate cognitive impairment has been shown to not even significantly influence the results of studies on preferences [29]. Therefore, how residents are involved needs to be adapted to their capabilities. The literature on stakeholder engagement recommends the use of supportive communication strategies to support vulnerable stakeholders, such as nursing home residents. These strategies involve the use of simple language, namely, short sentences, no more than one message per sentence, or frequently repeated words [47].

For this study, several methodological considerations need to be addressed. Representativity of the data might be compromised by the fact that only two teams from two different nursing homes in the Netherlands participated in this study. It is difficult to assess whether data saturation was reached due to the sample size. In addition, teams were monitored only after the evaluation meetings, which could have influenced the data saturation as well. To enrich the data, teams from different wards (psychogeriatric wards and somatic wards) were chosen, and perspectives of residents, family members and care professionals were included. The reliability of the data and the results were ensured by using a standardized observation form and detailed notes were taken during the process of data collection and analysis.

Further research should investigate how care teams, in collaboration with residents and family members, follow up after the first evaluation meeting and focus on what quality improvement strategies they implement and what the effects are on RCC. Therefore, an action research approach could be implemented to support the process of improving RCC through evidence-based knowledge of, for instance, team formation and collective learning.

Although recent literature suggests that narratives are a way to improve RCC, it seems necessary to conduct further research on this [40]. Moreover, it also seems necessary to further investigate the extent to which the depth of narrative data should be utilized to achieve meaningful quality improvement.

Based on the results of this study, several practical implications can be made. Narratives collected with Connecting Conversations can support RCC, as both taking time to have the conversations and using these stories for quality improvement foster deeper understanding, build trust, and create more personalized and meaningful care experiences. To use narrative data in quality improvement processes, a structured approach for evaluation meetings and a detailed preparation are necessary. Teams who start using the data need guidance to use the full range of details to implement deep learning strategies. This could be achieved by planning an extra scheduled meeting and organizing repetitive meetings, having a facilitator, ensuring a safe team climate (i.e., participant selection), and encouraging the reflective skills of the participants.

To facilitate family involvement in quality improvement processes, nursing home organizations should invest in building good family-caregiver relationships. Therefore, continuous dialog can be considered to communicate the expectations and preferences of both parties. Resident participation could be enhanced by using supportive communication strategies. If direct participation of residents and family members is not possible, indirect participation by a resident and family representative can also be considered.

## Conclusion

When using narrative quality data for quality improvement, the richness of the data is not used to its full potential. Discussions are focused on incidental problem solving rather than in-depth reflections on the meaning of events for providing RCC. Thus, improving RCC in collaboration with care professionals, family members and residents remains challenging. To improve RCC in collaboration with care professionals, residents and family members, a trusting relationship within the care triad first needs to be established.

## Data Availability

Research data supporting the findings of this study are not shared due to privacy or ethical restrictions.

## Abbreviation

QoC: Quality of Care
RCC: Relationship-centered care
